# Sensorimotor Reorganizations of Arm Kinematics and Postural Strategy for Functional Whole-Body Reaching Movements in Microgravity

**DOI:** 10.3389/fphys.2017.00821

**Published:** 2017-10-20

**Authors:** Thomas Macaluso, Christophe Bourdin, Frank Buloup, Marie-Laure Mille, Patrick Sainton, Fabrice R. Sarlegna, Jean-Louis Vercher, Lionel Bringoux

**Affiliations:** ^1^Aix Marseille Univ, CNRS, ISM, Marseille, France; ^2^UFR STAPS, Université de Toulon, La Garde, France; ^3^Department of Physical Therapy and Human Movement Sciences, Feinberg School of Medicine, Northwestern University, Chicago, IL, United States

**Keywords:** whole-body reaching, arm kinematics, postural strategy, sensorimotor adaptation, microgravity, parabolic flight, weightlessness

## Abstract

Understanding the impact of weightlessness on human behavior during the forthcoming long-term space missions is of critical importance, especially when considering the efficiency of goal-directed movements in these unusual environments. Several studies provided a large set of evidence that gravity is taken into account during the planning stage of arm reaching movements to optimally anticipate its consequence upon the moving limbs. However, less is known about sensorimotor changes required to face weightless environments when individuals have to perform fast and accurate goal-directed actions with whole-body displacement. We thus aimed at characterizing kinematic features of whole-body reaching movements in microgravity, involving high spatiotemporal constraints of execution, to question whether and how humans are able to maintain the performance of a functional behavior in the standards of normogravity execution. Seven participants were asked to reach as fast and as accurately as possible visual targets while standing during microgravity episodes in parabolic flight. Small and large targets were presented either close or far from the participants (requiring, in the latter case, additional whole-body displacement). Results reported that participants successfully performed the reaching task with general temporal features of movement (e.g., movement speed) close to land observations. However, our analyses also demonstrated substantial kinematic changes related to the temporal structure of focal movement and the postural strategy to successfully perform -constrained- whole-body reaching movements in microgravity. These immediate reorganizations are likely achieved by rapidly taking into account the absence of gravity in motor preparation and execution (presumably from cues about body limbs unweighting). Specifically, when compared to normogravity, the arm deceleration phase substantially increased. Furthermore, greater whole-body forward displacements due to smaller trunk flexions occurred when reaching far targets in microgravity. Remarkably, these changes of focal kinematics and postural strategy appear close to those previously reported when participants performed the same task underwater with neutral buoyancy applied to body limbs. Overall, these novel findings reveal that humans are able to maintain the performance of functional goal-directed whole-body actions in weightlessness by successfully managing spatiotemporal constraints of execution in this unusual environment.

## Introduction

On Earth, humans' motor behavior takes place within the ubiquitous gravitational force field. Several previous work already reported that the gravity direction and intensity are taken into account for motor execution, both on focal and postural components. For instance, regarding vertical arm movements, kinematic differences have been revealed between upward and downward movements (i.e., executed against or toward the direction of gravity). Particularly for upward arm movements, the relative deceleration duration was shown to be longer than the relative acceleration duration, while the opposite was observed for downward arm movements (Papaxanthis et al., 1998, 2003). Such asymmetric bell-shaped velocity profiles would allow humans to take advantage of mechanical effects of gravity torque on the limb by passively decelerating/accelerating upward/downward movements (Gaveau et al., 2014). This assumption is supported by the analysis of muscle activation patterns during vertical arm movements (Papaxanthis et al., 2003) and the removal of this specific asymmetry for horizontal movements wherein the gravitational torques did not vary (Gentili et al., 2007; Le Seac'h and McIntyre, 2007). Furthermore, these direction-dependent kinematic asymmetries appeared early in movement execution suggesting that the gravity effects could be anticipated and integrated into motor planning (Gaveau and Papaxanthis, 2011). Noticeably, the focal part of the movement investigated by these previous work is executed within a postural context, which was also subject to the influence of gravity. On Earth, body posture has to deal with the gravitational force to avoid falling. Indeed, humans would try to actively maintain the vertical projection of the center of mass (CoM) inside the support surface (Massion, 1992; Vernazza et al., 1996; Massion et al., 2004). Thus, trunk bending or upper limb movements may act as internal sources of disturbance to equilibrium. To prevent both substantial CoM displacement and falling, compensatory displacements of hip and knee usually occur (Babinski, 1899; Crenna et al., 1987; Massion, 1992; Horak, 2006).

Overall, studies mentioned above clearly demonstrated that the gravitational force plays an important role into the motor planning and execution on Earth. More precisely, the velocity profiles of arm movements and the postural strategy seem to be relevant gravity-dependent kinematic markers of human motor behavior. What happens however when gravity is removed? Understanding the impact of weightlessness on human behavior is of critical importance for keeping efficient sensorimotor behavior during the forthcoming long-term space missions. Parabolic and space flights contexts are privileged by researchers to investigate the effects of microgravity exposure on motor control. Previous studies focusing on arm movements revealed that final accuracy decreased in microgravity as compared to normogravity observations (Bock et al., 1992; Fisk et al., 1993; Watt, 1997; Carriot et al., 2004; Bringoux et al., 2012) which is consistent with works on pointing movements into a new force field (Lackner and DiZio, 1994; Shadmehr and Mussa-Ivaldi, 1994; Goodbody and Wolpert, 1998; Bourdin et al., 2001, 2006; Lefumat et al., 2015). However, the way microgravity exposure impacts kinematic features remains unclear. Indeed, some authors observed a reduction of movement speed (Ross, 1991; Berger et al., 1997; Mechtcheriakov et al., 2002; Carriot et al., 2004; Crevecoeur et al., 2010) whereas others reported no significant changes as compared to normogravity (Papaxanthis et al., 2005; Bringoux et al., 2012; Gaveau et al., 2016). More interestingly, contrasting findings have been also reported concerning gravity-dependent kinematic markers based on the temporal organization of focal movement and postural behavior. Indeed, some studies of arm vertical movements performed during parabolic flights showed either a progressive disappearance of asymmetric velocity profiles (Papaxanthis et al., 2005; Gaveau et al., 2016) or conversely an increase of the relative deceleration duration (Bringoux et al., 2012) with respect to normogravity. Regarding postural control in microgravity, most previous work demonstrated the persistence of a terrestrial strategy by stabilizing the CoM displacements during internal disturbance, such as trunk bending or arm and leg raising (Massion et al., 1993, 1997; Mouchnino et al., 1996; Vernazza-Martin et al., 2000). However, during long-term exposure, Pedrocchi et al. (2002, 2005) reported significant shifts of CoM toward the moving leg on a same lateral lower limb raising task.

In these previous experiments, it should be noted that the focal and postural parts of movement were separately investigated, although both components are known to largely interact during functional motor behavior. Only few works have studied goal-directed whole-body reaching movements in microgravity and contradictory findings were reported. On the one hand, Patron et al. (2005) reported a decrease of the relative deceleration duration of arm movement associated to a stabilized CoM displacement in microgravity. On the other hand, Casellato et al. (2012) reported an invariance of the asymmetry of the hand velocity profile as compared to normogravity data, associated to a vertical CoM projection beyond the base of support. These discrepant findings may partly originate from inter-individual variability, as Casellato et al. (2016) recently observed different and highly variable behaviors regarding CoM stabilization on three astronauts onboard the ISS (long-term exposure). Most importantly, task-related concerns, especially target location, body limbs displacements and movement speed, could also explain these contradictory results. For instance, Patron et al. (2005) investigated postural influences on a reaching task toward targets close to the participant's feet with or without speed instructions, while Casellato et al. (2012) asked the participants to perform unconstrained forward hand movements toward targets located beyond arm's length. However, to the best of our knowledge, we are not aware of any study which has investigated goal-directed whole-body reaching movements requiring to be performed as fast and as accurate as possible in microgravity.

The present study thus aimed at characterizing kinematic features of goal-directed whole-body reaching movements in microgravity, involving high spatiotemporal constraints of execution, by comparing them to normogravity observations. The spatial requirements were defined in terms of target location and size, while the temporal requirements referred to the necessity of performing the movements as fast as possible within the accuracy constraints. To that aim, close versus far external visual targets were presented during microgravity episodes in parabolic flight. To reach far targets, additional whole-body displacement was required. For both targets, two different sizes of target area were presented. As indicated by studies mentioned above, task requirements must be accounted for when considering the impact of microgravity on motor behavior. Thus, the high spatiotemporal constraints of execution in the present study constitute a novel approach allowing us to investigate whole-body reaching movements through a more functional behavior in weightless environments, close to those performed by astronauts during their space missions. In other words, we question whether and how humans are able to maintain the performance of a functional behavior in the standards of normogravity execution. We predicted substantial changes of gravity-dependent kinematic markers reflecting the specific reorganizations of focal and postural components in microgravity as compared to normogravity.

## Materials and methods

### Participants

Seven right-handed (3 women and 4 men, mean age = 39 ± 6.9 years) participated in the experiment on a voluntary basis. They had no prior experience of microgravity exposure. As the present study is part of a scientific program studying human motor behavior in different force fields, participants were previously tested in normogravity and underwater for the same task as reported in Macaluso et al. (2016). None of the participants suffered from neuromuscular or sensory impairments, as confirmed by a medical examination prior to the experiment. Vision was normal or corrected by lenses. Before microgravity exposure, the participants received comfort medication (scopolamine) to avoid motion sickness. It has been demonstrated that its use for parabolic flights did not induce neuromuscular side-effects on sensorimotor control (Ritzmann et al., 2016). All the participants were naive as to the specific purpose of the experiment, which was authorized by the ANSM (French National Agency for Biomedical Security) and approved by the Committee for the Protection of Persons concerned (CPP). The participants gave their signed informed consent prior to the study in accordance with the Helsinki Convention.

### Experimental setup

**Figure 1:**
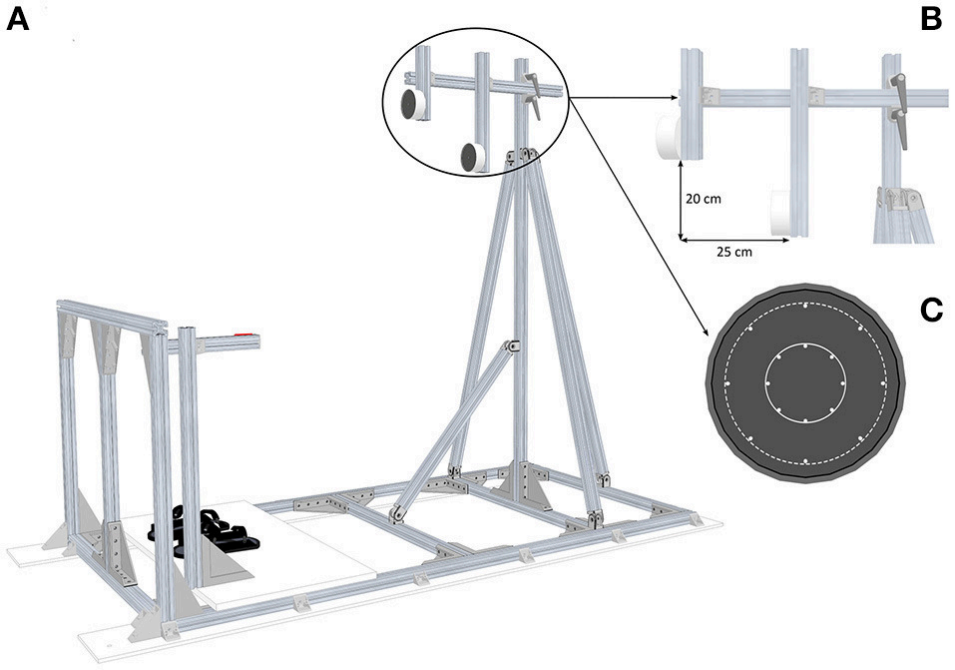
Experimental setup. **(A)** Global view of the pointing structure including targets, start push-button and footstraps. **(B)** Side view of the targets which illustrates the position of the Far targets relative to the Close targets. **(C)** Front view of the two target sizes: the solid line area represents the Small targets (Ø 4 cm) and the dotted line area represents the Large targets (Ø 10 cm).

Circular targets were presented in front of participants standing upright and maintained to the ground structure by means of foot-straps (Figure 1A). They had to press their right index finger on the start push-button positioned alongside their body. The height of the start push-button was adjusted to each participant's height for initial posture standardization. Circular targets were oriented along the frontal plane and were positioned relative to participants' anthropometric features. Close targets were located at shoulder's height (i.e., the height of the target center corresponded to the horizontal projection of the height of the acromioclavicular joint in the sagittal plane) at a distance corresponding to arm length, allowing the participants to reach these targets without trunk displacement. Far targets were located 25 cm away and 20 cm below the close targets: in that case, participants had to perform additional trunk displacement to reach these targets (Figure 1B). For both target locations, the diameter was also manipulated through Light-Emitting Diodes (LEDs) equally distributed to define two target sizes: Small targets 4 cm or Large targets 10 cm (Figure 1C). Therefore, in this experiment, combining location and size corresponded to the presentation of four targets: CS (Close–Small), CL (Close–Large), FS (Close–Small), FL (Far–Large). Switching targets on and off were achieved by a homemade software (Docometre©) piloting a real-time acquisition/control system running at 10 kHz (ADwin-Gold, Jäger, Lorsch, Germany).

Markers were positioned onto the participants' index, shoulder and hip. Markers position was recorded (i) in normogravity with a video motion capture system (LED-type markers) composed of three cameras sampled at 60 Hz (resolution: 848 × 480 pixels); (ii) in microgravity by an optical motion capture system (infra-red active markers) at 200 Hz (Codamotion CXS and Active CodaHub, Charnwood Dynamics Ltd, Leicestershire, UK). Importantly, both acquisition systems yielded a similar accuracy in the definition of markers' position. Indeed, the data acquired in NormoG by the video motion capture system were processed using Direct Linear Transformation (Abdel-Aziz and Karara, 1971), to reach the accuracy level of the optical motion capture system used in MicroG (i.e., millimeter order). Moreover, according to the sampling theorem (Shannon, 1949), the sampling rates used in both environments are known to be sufficient to capture the whole range of velocities associated to biological motion, including fast reaching movements (Song and Godøy, 2016).

### Procedure

All participants were exposed to two environments: first in normogravity (“NormoG”) before the parabolic flight campaign, then in microgravity (“MicroG”). The MicroG environment was achieved in the A-310 ZERO-G aircraft chartered by the French Centre National d'Etudes Spatiales (CNES) and Novespace for parabolic flight studies, during the campaign #125, including 3 days of flight. For each flight, the aircraft ran a sequence of 30 parabolas. Parabolic maneuver was composed of three distinct phases: 24 s of hypergravity (1.8 g, pull-up phase) followed by 22 s of microgravity (0 g) before a second period of 22 s of hypergravity (1.8 g, pull-out phase). Each parabola was separated by 1 min of normogravity (1 g, steady flight phase).

Positions of the start push-button and the targets were adjusted for each participant, then an initial calibration of targets was performed along the Z vertical axis (i.e., defining positions relative to arm movement elevation). Before each trial, participants had to stand upright, the arms outstretched along the body, and the right index pressing the start push-button. When one of the targets was illuminated, participants were asked to perform a reaching movement toward the target while keeping the arm outstretched. Reaching movements had to be performed as fast as possible while primarily respecting accuracy constraints related to the target area. Each trial was validated when the index fingertip reached the target. The final position had to be maintained until target extinction (3 s after movement onset) which prompted the participants to return to the starting position.

Participants performed 10 pointing movements toward each of the four targets for a total of 40 trials per experimental session in each environment. In the MicroG environment, these 40 trials were presented during 10 successive parabolas for each participant, thus including four trials per parabola. The targets were presented in a pseudorandom order, which was counterbalanced between the participants. Each session included three specific blocks of four trials in which the order of target presentation was the same. These blocks were presented in the initial, middle and final part of the session (corresponding to the 1, 5, and 10th parabola in MicroG) to assess the potential evolution of motor performance during each session, which lasted about 25 min.

### Data processing

Data presented below describe behavioral features of reaching movements in the sagittal plane and some of them are detailed in Macaluso et al. (2016). First, we analyzed the fingertip trajectory, success rate (index fingertip within a given target area), index final deviation from target center, reaction time (RT), movement duration (MD), and mean tangential velocity (Vmean_endpoint_). The index final deviation was measured as the mean absolute distance of the final position of the index fingertip relative to the target center along the Z vertical axis. For each trial, the time elapsed between target illumination and the release of the start push-button by the participants defined RT. Index position in the sagittal plane was filtered (digital second-order dual-pass Butterworth filter; cutoff frequency 6 Hz in NormoG and 10 Hz in MicroG) and differentiated to obtain the endpoint tangential velocity in m.s^−1^. Regarding the different sampling rates of acquisition systems used in both environments, we found that the cutoff frequencies mentioned above were the most suitable to reflect the raw data in normo and microgravity. The movement onset was defined as the time when the index tangential velocity reached 1.5% of its peak. Conversely, movement end was defined when the tangential velocity dropped below 1.5% of its peak.

The focal component of whole-body reaching movements was analyzed by considering the arm angular elevation over time (i.e., angle evolution of the extended arm around the shoulder with respect to its initial orientation). Arm angular elevation was computed from the index and shoulder XZ raw data, filtered (digital second-order dual-pass Butterworth filter; cutoff frequency 6 Hz in NormoG and 10 Hz in MicroG) and differentiated to obtain the arm angular velocity profile. From this velocity profile, the peak velocity (PV_ang_ in deg.s^−1^) and the relative angular deceleration duration (rDD_ang_, defined as the duration between PV_ang_ and movement end, expressed in % of movement duration to facilitate comparison between both environments) were extracted. Arm angular velocity profile was also differentiated to obtain arm angular acceleration profile, informing on early changes in motor execution which may give an insight upon the planning stage of focal movement. From this acceleration profile, peak acceleration (PA_ang_ in deg.s^−2^) and time to peak acceleration (TPA_ang_ expressed in ms to precisely estimate the occurrence of motor changes) were extracted.

In parallel, the postural component involved in the whole-body reaching movements (particularly to reach the far target) was analyzed by considering trunk displacement. This latter was illustrated by the final angular position of trunk (hip-shoulder segment) relative to vertical (β_f_trunk: trunk flexion in deg) at arm movement end, and by the forward displacement of participants' shoulder and hip (translation along the horizontal plane in mm). Shoulder and hip movement onset/end in the sagittal plane were defined as the time when the tangential velocity respectively reached/dropped below 1.5% of its peak.

Statistical analyses were based on mean comparisons. Repeated-measures analyses of variance (ANOVAs) were performed to compare the means of kinematic parameters mentioned above after having ensured that the assumption of normality was not violated (Kolmogorov-Smirnov test). Newman-Keuls tests were used for *post-hoc* analyses and the significance threshold was set at.05 for all statistical tests.

## Results

### Potential learning effects

We conducted prior analyses to investigate the potential learning effects during a single session (40 trials). Repeated-measures ANOVAs including 2 Environment (NormoG, MicroG) × 2 Target Location (Close, Far) × 2 Target Size (Small, Large) × 3 Block (Initial, Middle, Final) were initially performed on all the selected parameters of the study. The results did not show any significant main effect of Block or any interaction with the other factors (*p* > 0.05). To specifically exclude the presence of any adaptive processes in MicroG environment, we conducted complementary analyses comparing a specific set of trials occurring during the 1, 5, and 10th parabola (see Material and Methods). Repeated-measures ANOVAs including 3 Parabola (1, 5, and 10th parabola) × 4 Target Presentation (CS, CL, FS, FL) did not reveal any significant main effect of Parabola or any interaction with the other factor on all the selected parameters (*p* > 0.05). Thus, the reported values did not significantly change throughout the experiment.

### Upper-limb displacement

**Figure 2:**
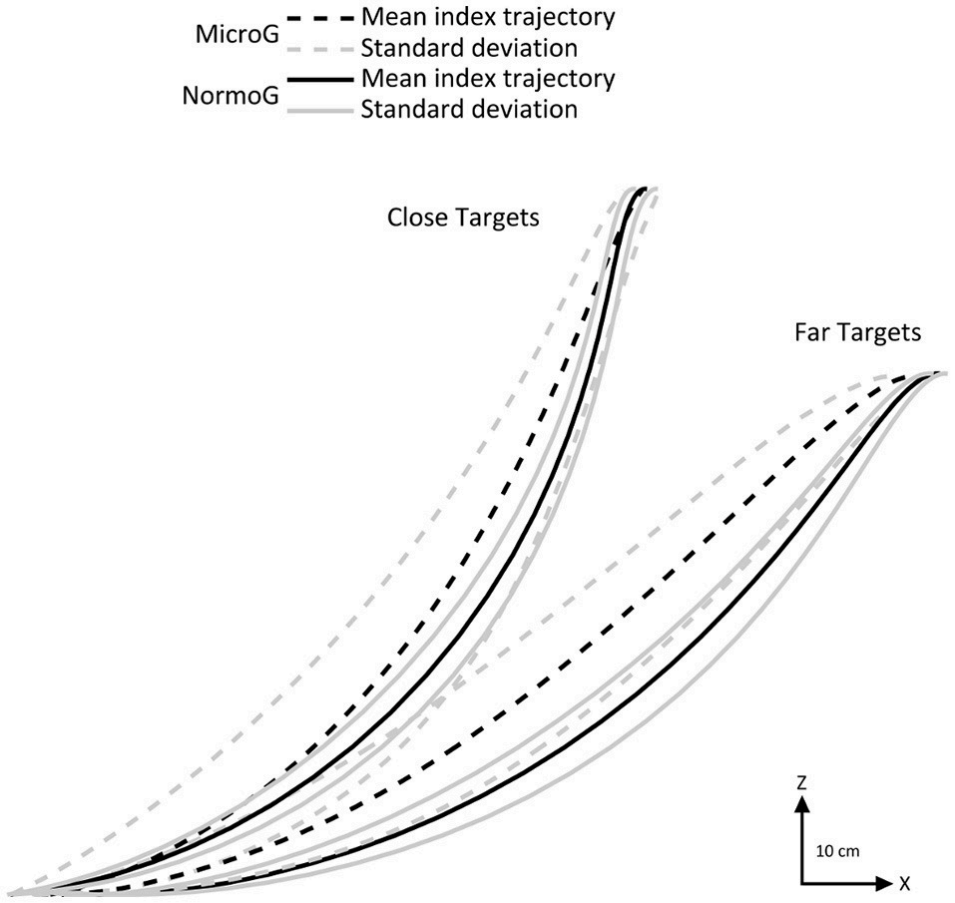
Representative mean endpoint trajectories (black lines) for a typical subject in the sagittal plane in MicroG (dotted line), and NormoG (solid line) for the Close and Far targets. Gray lines represent the positive and negative standard deviations of the mean index trajectories.

First of all, we investigated arm movement toward the targets in each environment. Figure 2 illustrates mean endpoint trajectories (i.e., index fingertip) in the sagittal plane observed for a typical participant when reaching close and far targets. It shows that spatial characteristics of endpoint motion were impacted by the microgravity environment.

#### Success rate and index final deviation

Overall, participants successfully performed the task. Indeed, success rate was 100% in NormoG and 95.42 ± 8.99% in MicroG. In this latter environment, only the Small targets were sometimes missed (CS and FS). The ANOVA performed on success rate revealed no significant main effect of the experimental conditions (Environment: *p* = 0.06; Target Location: *p* = 0.39; Target Size: *p* = 0.06) and no significant interaction between these factors (Environment × Target Location: *p* = 0.39; Environment × Target Size: *p* = 0.06; Target Location × Target Size: *p* = 0.39). Moreover, the ANOVA conducted on the index final deviation yielded no main effect of the experimental conditions (Environment: *p* = 0.10; Target Location: *p* = 0.97; Target Size: *p* = 0.06) but showed a significant interaction between Environment × Target Size [*F*_(1, 6)_ = 8.49; *p* < 0.05]. While no significant difference appeared between both environments when reaching Small targets (*p* > 0.05), the mean distance between the final position of the index and the target center when reaching Large targets was significantly higher in MicroG as compared to NormoG (13.04 ± 6.07 mm vs. 7.41 ± 2.96 mm; *p* < 0.01). No significant interaction between the other factors was revealed (Environment × Target Location: *p* = 0.69; Target Location × Target Size: *p* = 0.32).

#### Reaction time (RT)

The ANOVA performed on RT (mean = 326 ± 70 ms) revealed no significant main effect of the experimental conditions (Environment: *p* = 0.48; Target Location: *p* = 0.23; Target Size: *p* = 0.43) and no significant interaction between these factors (Environment × Target Location: *p* = 0.19; Environment × Target Size: *p* = 0.23; Target Location × Target Size: *p* = 0.52).

#### Movement duration (MD) and mean tangential velocity (Vmean_endpoint_)

The ANOVA conducted on MD only yielded a significant main effect of Target Location [*F*_(1, 6)_ = 166.21; *p* < 0.001]; MD was longer when reaching Far targets (0.73 ± 0.17 s) as compared to Close targets (0.58 ± 0.16 s). No other significant main effect or interaction was found with regard to the other factors (Environment: *p* = 0.07; Target Size: *p* = 0.11; Environment × Target Location: *p* = 0.35; Environment × Target Size: *p* = 0.26; Target Location × Target Size: *p* = 0.59).

The ANOVA conducted on Vmean_endpoint_ revealed significant main effects of Target Location [*F*_(1, 6)_ = 24.05; *p* < 0.01] and Target Size [*F*_(1, 6)_ = 11.30; *p* < 0.05]. Vmean_endpoint_ was higher when reaching Close targets (1.94 ± 0.39 m.s^−1^ vs. 1.66 ± 0.31 m.s^−1^, respectively for Close and Far targets). Vmean_endpoint_ was also higher when reaching Large targets (1.83 ± 0.39 m.s^−1^ vs. 1.76 ± 0.37 m.s^−1^, respectively for Large and Small targets). No other significant main effect or interaction was found with regard to the other factors (Environment: *p* = 0.52; Environment × Target Location: *p* = 0.14; Environment × Target Size: *p* = 0.76; Target Location × Target Size: *p* = 0.91).

To sum up, microgravity did not significantly affect the performance of whole-body reaching movements without substantially disrupting the general temporal outputs of endpoint displacement and the success rate. Then, we investigated the temporal organization of the focal component illustrated by the arm angular elevation over time.

#### Temporal organization of arm angular elevation

**Figure 3:**
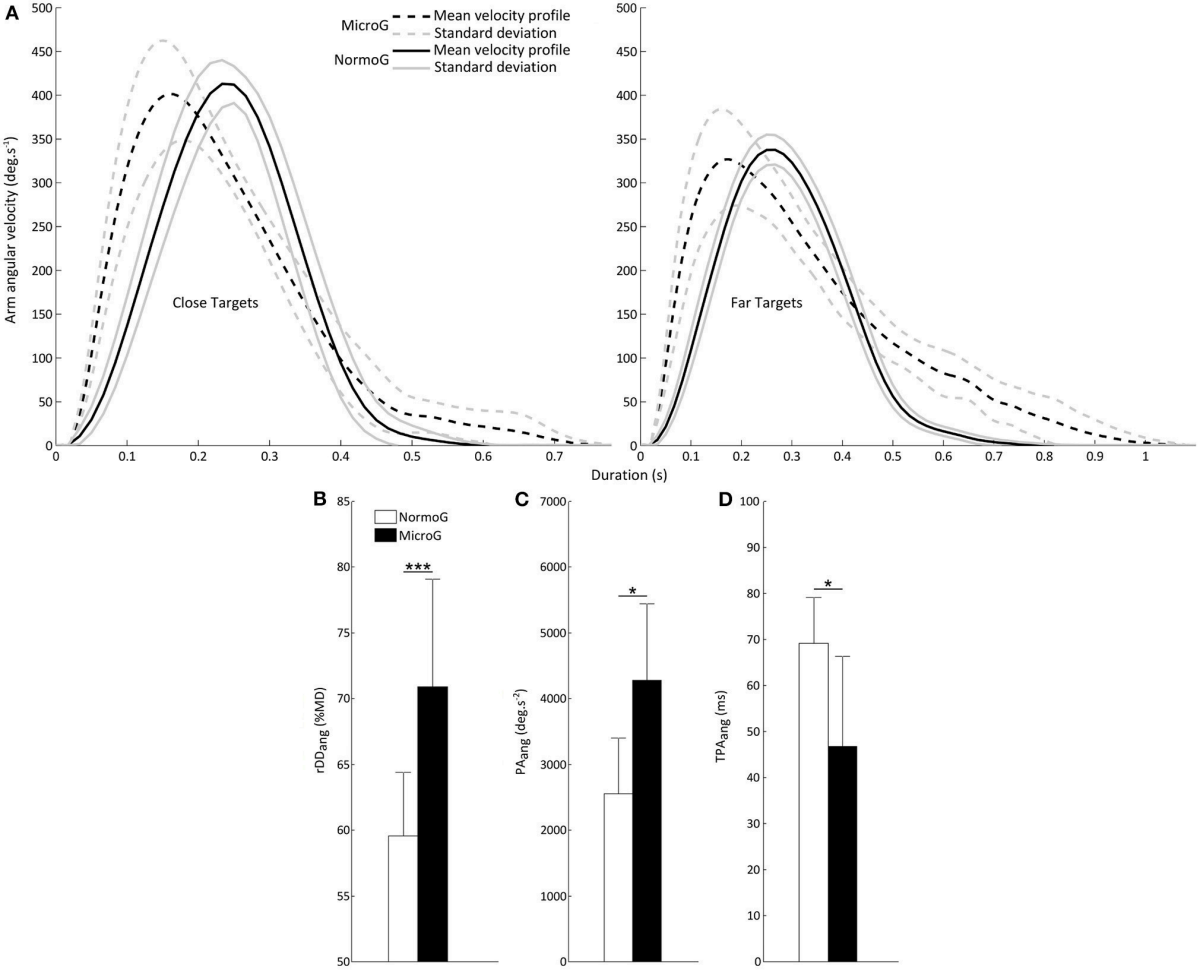
**(A)** Representative mean arm angular velocity profiles for a typical subject in MicroG (dotted line) and NormoG (solid line) for the Close and Far targets. Gray lines represent the positive and negative standard deviations of the mean arm angular velocities. **(B)** Mean relative angular deceleration duration (rDD_ang_) as a function of Environment. **(C)** Mean peak angular acceleration (PA_ang_) and **(D)** Mean time to peak angular acceleration (TPA_ang_) as a function of Environment. Error bars represent standard deviation of the mean. ^***^p < 0.001; ^*^p < 0.05.

Figure 3A illustrates mean arm angular velocity profiles for a typical participant when reaching Close and Far targets in each environment. It shows that the MicroG environment impacts the temporal structure of the velocity profile [reflected by the analysis of *rDD*_*ang*_, see below Velocity profile: peak angular velocity (PV_ang_) and relative angular deceleration duration (rDD_ang_)] without substantially changing its amplitude. As reported below, this modulation could derive from changes of the temporal structure and amplitude of the acceleration profile [as suggested by the analysis of *TPA*_*ang*_ and *PA*_*ang*_, see below Acceleration profile: peak angular acceleration (PA_ang_) and time to peak angular acceleration (TPA_ang_)].

##### Velocity profile: peak angular velocity (PV_ang_) and relative angular deceleration duration (rDD_ang_)

The ANOVA conducted on PV_ang_ only revealed a significant main effect of Target Location [*F*_(1, 6)_ = 58.74; *p* < 0.001]. PV_ang_ was higher when reaching Close targets (397.98 ± 68.77 deg.s^−1^) as compared to Far targets (327.55 ± 48.39 deg.s^−1^). No other significant main effect or interaction was found with regard to the other factors (Environment: *p* = 0.29; Environment × Target Location: *p* = 0.08; Environment × Target Size: *p* = 0.73; Target Location × Target Size: *p* = 0.51).

The ANOVA conducted on rDD_ang_ revealed significant main effects of Environment [*F*_(1, 6)_ = 48.54; *p* < 0.001], Target Location [*F*_(1, 6)_ = 20.91; *p* < 0.01] and Target Size [*F*_(1, 6)_ = 7.38; *p* < 0.05]. Importantly, rDD_ang_ was substantially higher in MicroG as compared to NormoG (Figure 3B). Overall, rDD_ang_ was higher when reaching Far targets (69.65 ± 7.69%MD vs. 60.80 ± 7.63%MD, respectively for Far and Close targets) and Small targets too (65.63 ± 9.05%MD vs. 64.83 ± 8.71%MD, respectively for Small and Large targets). No significant interaction was found between these factors (Environment × Target Location: *p* = 0.22; Environment × Target Size: *p* = 0.54; Target Location × Target Size: *p* = 0.44).

##### Acceleration profile: peak angular acceleration (PA_ang_) and time to peak angular acceleration (TPA_ang_)

The ANOVA performed on PA_ang_ revealed significant main effects of Environment [*F*_(1, 6)_ = 9.30; *p* < 0.05] and Target Location [*F*_(1, 6)_ = 73.70; *p* < 0.001]. PA_ang_ was higher in MicroG than NormoG (Figure 3C) and also higher when reaching Close targets (3661.18 ± 1332.30 deg.s^−2^) as compared to Far targets (3175.85 ± 1265.36 deg.s^−2^). No other significant main effect or interaction was found with regard to the other factors (Target Size: *p* = 0.54; Environment × Target Location: *p* = 0.23; Environment × Target Size: *p* = 0.99; Target Location × Target Size: *p* = 0.98).

The ANOVA conducted on TPA_ang_ also yielded significant main effects of Environment [*F*_(1, 6)_ = 7.43; *p* < 0.05] and Target Location [*F*_(1, 6)_ = 8.92; *p* < 0.05). Importantly, TPA_ang_ was lower in MicroG than in NormoG (Figure 3D) and also lower when reaching Far targets (54 ± 18 ms) as compared to Close targets (62 ± 20 ms). No other significant main effect or interaction was found with regard to the other factors (Target Size: *p* = 0.06; Environment × Target Location: *p* = 0.92; Environment × Target Size: *p* = 0.42; Target Location × Target Size: *p* = 0.95).

In summary, microgravity exposure influenced the temporal structure of arm angular elevation by decreasing the time to peak acceleration, thus leading to an increase of the relative deceleration duration as compared to NormoG. These modifications did not affect the maximal velocity of arm elevation in MicroG as compared to NormoG, presumably because of a higher maximal acceleration reached earlier during movement execution. The next part will focus on the postural component involved in whole-body reaching movements, particularly when reaching Far targets.

### Trunk displacement

#### Final angular position of trunk relative to vertical (β_f_trunk)

**Figure 4:**
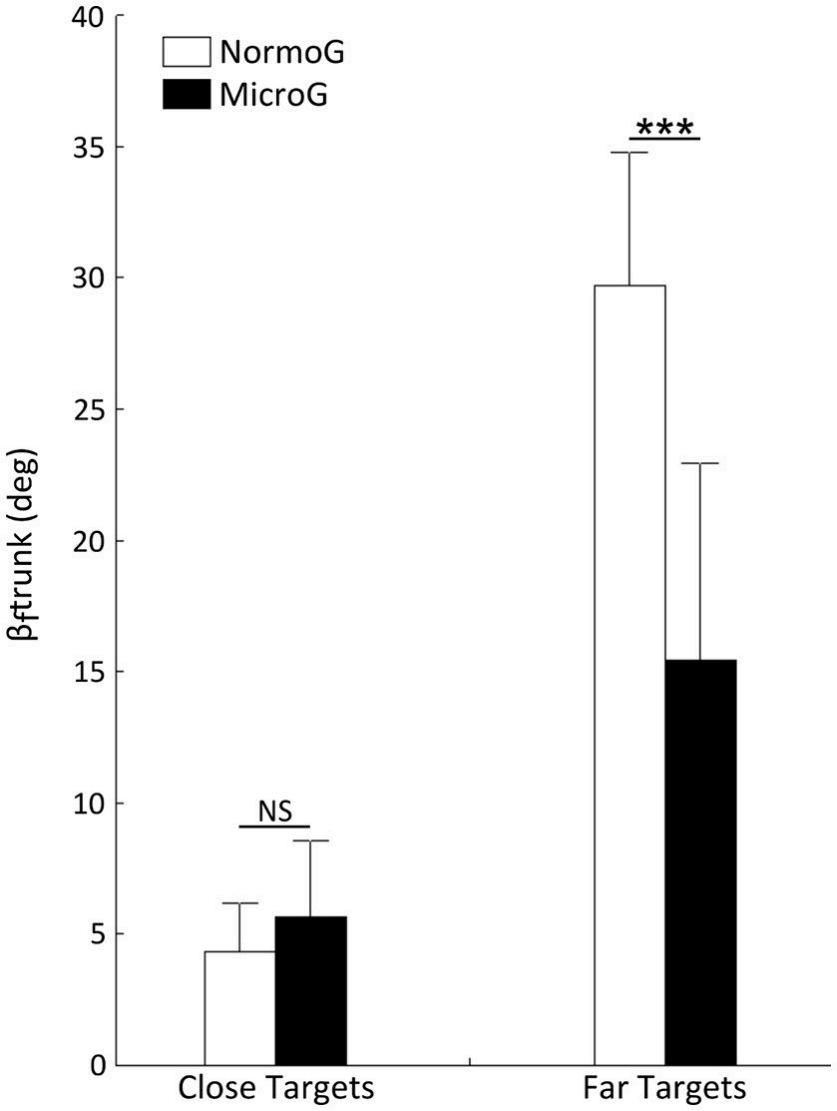
Mean final angular position of trunk relative to vertical (β_f_trunk) as a function of Environment and Target Location. Error bars represent standard deviation of the mean. ^***^p < 0.001; NS, non-significant difference.

The ANOVA performed on β_f_trunk revealed a main effect of Target Location [*F*_(1, 6)_ = 264.09; *p* < 0.001] and a significant interaction between Environment × Target Location [*F*_(1, 6)_ = 24.74; *p* < 0.01]. Interestingly, while no significant difference appeared between both environments when reaching Close targets (*p* > 0.05), mean β_f_trunk was significantly lower when reaching Far targets in MicroG as compared to NormoG (*p* < 0.001; Figure 4).

#### Shoulder and hip forward displacement

Unsurprisingly in both environments, no noticeable forward translation was detected for shoulder and hip when reaching Close targets (located at participants' arm length, see Material and Methods). Therefore, we subsequently led our analysis on the shoulder and hip forward displacement occurring when reaching Far targets.

The ANOVA conducted on shoulder displacement yielded a significant main effect of Environment [*F*_(1, 6)_ = 183.78; *p* < 0.001]. Shoulder displacement in MicroG (448.28 ± 25.37 mm) was significantly higher than in NormoG (285.54 ± 36.18 mm). The ANOVA performed on hip displacement revealed significant main effects of Environment [*F*_(1, 6)_ = 20.94; *p* < 0.01] with higher displacement in MicroG (185 ± 84.28 mm) as compared to NormoG (38.68 ± 39.49 mm). The ANOVA also revealed a main effect of Target Size [*F*_(1, 6)_ = 9.09; *p* < 0.05] and a significant interaction between Environment × Target Size [*F*_(1, 6)_ = 7.34; *p* < 0.05]. While no significant difference appeared between Small and Large targets in NormoG, mean hip displacement in MicroG was higher when reaching Large target (191.66 ± 86.15 mm) as compared to Small target (178.50 ± 88.70 mm).

Overall, these analyses highlight that the postural component varied during whole-body reaching movements mainly as a function of the Environment and Target Location. In MicroG, reaching Far targets involved smaller trunk bending associated to larger forward displacements of the shoulder and hip, as compared to NormoG. In the next section, we will discuss the main focal and postural features reported above and will propose possible interpretations for these observations.

## Discussion

The present study aimed at characterizing kinematic features of goal-directed whole-body reaching movements in microgravity involving high spatiotemporal constraints of execution, with respect to normogravity observations. Our original experimental design enabled us to investigate reaching movements performed as fast as possible toward targets of different sizes and locations in both environments. Our data revealed stabilized motor features throughout microgravity exposure. While some of them are associated to the preservation of general temporal outputs with respect to land observations (e.g., movement speed), we found substantial changes in gravity-dependent kinematic markers reflecting the reorganization of focal and postural components. These points will be developed in the following sections.

### Prompt reorganization of motor behavior in microgravity

Although the participants never experienced microgravity exposure before the present experiment, we did not find any significant evolution in the reported variables across the successive trials. Thus, we failed to show the presence of sensorimotor adaptation during the experiment which would indeed have led to more progressive changes across the repetition of reaching movements (Lackner and DiZio, 1994; Shadmehr and Mussa-Ivaldi, 1994). Rather, we observed a prompt reorganization of some movement features (see section Microgravity Is Accounted for into the Planning of Focal Movement) which took place at the earliest onset of exposure. From previous work conducted in parabolic flights, the occurrence of adaptive processes on reaching movements is not clear. Indeed, some studies reported slow progressive changes of kinematics across parabolas (Papaxanthis et al., 2005; Gaveau et al., 2016) whereas others observed rapid behavioral stabilization or no significant change during exposure (Patron et al., 2005; Bringoux et al., 2012; Casellato et al., 2012). In our study, one hypothesis related to the parabolic flight context can be advanced to explain this immediate stabilization of motor behavior. Before the 30 parabolas achieved for experimental acquisition, the aircraft performed one parabola to allow participants discover the parabolic maneuver. Moreover, since 3 participants were tested during each flight (see Material and Methods), two of them had even more time to experience microgravity exposure. Although we ensured that no reaching movements were performed by the participants out of the experiment, this preliminary although short exposure before data acquisition would enable the participants to develop prior expectancies about how it feels to move in these novel environments. Moreover, microgravity episodes of parabolic flights induced a global modification of the force field applied to the whole-body before initiating each trial. Thus, in this context, the participants accessed the new dynamic properties of the environment prior to movement onset (Barbiero et al., 2017) which might be sufficient to rapidly update their internal model for sensorimotor planning and execution, hence leading to an immediate motor reorganization (Wolpert and Kawato, 1998; Wolpert and Ghahramani, 2000). It has been shown indeed that the initial state of the sensorimotor system is primarily used to adjust the internal representations necessary to perform upcoming movements (Starkes et al., 2002; Flanagan et al., 2006; White et al., 2012, Rousseau et al., 2016). Here, the limb proprioception could contribute to detect the gravity release at the level of muscles and joints, through muscle spindles and Golgi tendon organs identified as load receptors related to gravity force field (Dietz et al., 1992). The following sections aim at discussing the stabilized motor features observed in microgravity for whole-body reaching.

### Preservation of functional reaching movements within normogravity standards

The high spatiotemporal constraints of execution in the present study enabled us to investigate functional whole-body reaching movements in microgravity. In this line, our data did not reveal any significant difference between MicroG and NormoG environments in terms of movement duration, mean and peak velocity during movement execution. The absence of effect of the environment on these variables may reflect a tendency to keep the average movement speed in the range of normogravity experience. To that aim, the participants may have reduced the safety margin related to the final reaching accuracy in microgravity. Indeed, while the preservation of movement speed was not detrimental to reaching performance (i.e., the high success rate observed in MicroG, > 95%, was not significantly different from NormoG), the distance between the endpoint final position and the target center was significantly higher in MicroG when reaching large targets. Thus, in this task, the participants tended to maintain speed over accuracy margin (Woodworth, 1899) for a still successful performance without gravity. Keeping the average speed and reaching performance within normogravity standards were though not at the expense of movement preparation duration, since the reaction time remained also unaffected by the environment. Hence, alleviating gravity before movement execution did not impact the time allocated for motor planning. Nevertheless, we will detail in the following parts some evidence for substantial qualitative reorganizations, notably in focal and postural components of the reaching movement, which helped maintain the functionality of motor behavior in microgravity.

### Microgravity is accounted for into the planning of focal movement

On Earth, kinematics of arm movement elevation has been well-described in terms of asymmetric bell-shaped velocity profiles (Papaxanthis et al., 1998; Gentili et al., 2007). Classically, the relative deceleration duration appears longer than the relative acceleration duration, suggesting that gravity is accounted for during motor planning to act as an assistive force for decelerating upward movements (Papaxanthis et al., 2003; Gaveau and Papaxanthis, 2011). The way gravity is integrated into motor planning has been recently formalized by a Minimum Smooth-Effort model (Gaveau et al., 2016), in line with the optimal control theory minimizing absolute work and jerk (Berret et al., 2011; Gaveau et al., 2014).

In our experiment, the substantial increase of the relative deceleration duration in MicroG constitutes the most salient feature of motor reorganization concerning the focal part of the reaching movement. The asymmetry was thus notably amplified in microgravity without changing the amplitude of peak velocity. Such reorganization, consistent across subjects as shown in the Supplementary Figure 1A, appears as a direct consequence of an earlier peak acceleration observed during motor execution (~47 ms). Recent work established that the shortest feedback-based corrections of EMG (electromyographic) patterns during arm reaching occur at ~60 ms when considering a limb disturbance with no changes of target location (Scott, 2016 for a review). We therefore hypothesize that the kinematic changes promptly observed in microgravity following arm movement onset are based on feedforward control mechanisms, directly expressed in the motor intention (Gaveau and Papaxanthis, 2011). In other words, we argue that the CNS could predict the effect of gravity release on the moving segments and could subsequently integrate the novel dynamics associated to a weightless environment into motor planning. As there is no external force to help braking upward movements in microgravity (neglecting the air friction forces), the participants had to actively counteract the inertial force of their moving limbs, presumably by increasing the antagonist muscle activations (Bonnard et al., 1997). In this context, a longer deceleration phase may reflect a greater use of feedback processes (Chua and Elliott, 1993; Sarlegna et al., 2003; Terrier et al., 2011). This greater retroactive control would enable the participants to better manage the speed reduction of their reaching movements, especially when approaching the target, to maintain final accuracy. Increased asymmetry between acceleration and deceleration phases was also reported when removing gravitational shoulder torque before arm movement onset (Rousseau et al., 2016) and with additional loads placed on the arm (Gaveau et al., 2011). Such reorganization in kinematics may thus illustrate a cautious strategy accounting for force/inertia uncertainties in unusual context. In this line, previous studies demonstrated that the lack of information prior to movement onset strongly affects motor planning (Bringoux et al., 2012; Rousseau et al., 2016), presumably to face unexpected or erroneous sensorimotor estimates during subsequent movement execution in unfamiliar environments (Brooks et al., 2015).

Contradictory with the present findings, some other studies reported a progressive disappearance of asymmetric velocity profiles in microgravity (Papaxanthis et al., 2005; Gaveau et al., 2016). Unlike our experiment, movement accuracy was not a primary constraint in these previous work where the braking phase of arm movements was not crucial during motor execution to correctly perform the task. Alternatively, when the participants had to perform reaching movements “*as accurately as possible*” in microgravity, Bringoux et al. (2012) also observed a longer deceleration phase as compared to normogravity exposure. Interestingly in our study, such reorganization of motor planning for arm reaching in MicroG was not detrimental to movement duration: the longer deceleration phase was compensated by higher peak acceleration in microgravity. This compensatory increase of peak acceleration may also represent a specific reorganization of the movement in a given environment as it likely exploits the absence of gravity torque at movement onset to efficiently trigger the initial impulse. Additionally, as discussed in the following section, the planning of focal movement was not the only component to be modified during whole-body reaching movements in microgravity.

### Efficient postural strategy for reaching without gravity

Kinematics collected from the trunk clearly supports two different postural strategies as a function of the gravity environment while reaching far targets placed beyond arm's length. Under normogravity, our analyses revealed a significant forward trunk bending expressed by large shoulder displacement associated to very small hip displacement in space. This feature is typical of a “*hip strategy*” (Horak and Nashner, 1986), through which the postural component supporting the focal part of movement is also used to prevent falling (Massion, 1992). This posturokinetic organization would thus reduce the displacement of the CoM by using compensatory mechanisms (Massion, 1992; Vernazza et al., 1996) and would favor equilibrium maintenance at the expense of mechanical energy minimization and joint smoothness maximization (Hilt et al., 2016).

Alternatively, the second postural strategy specifically observed in microgravity was illustrated by very small trunk bending associated to larger shoulder and hip displacement from vertical. This organization would resemble the “*ankle strategy*” evoked by Horak and Nashner (1986), though with greater whole-body forward displacement. In MicroG environment, the participants were indeed not constrained by gravitational force, allowing for a vertical CoM projection outside the base of support. This observation is consistent with others reporting that postural control in weightlessness is predominantly managed at the ankle level (Clement et al., 1984; Clément and Lestienne, 1988). On Earth, this posturokinetic strategy decreases the equilibrium safety margin but the risk of falling is greatly minimized in microgravity. The participants might therefore adopt the strategy which would allow them to reduce the degrees of freedom (Bernstein, 1967), helping minimize the mechanical energy expenditure and maximize joint smoothness (Hilt et al., 2016). In line with the optimal control theory (Berret et al., 2011; Gaveau et al., 2014), the combination of these cost functions would enable the postural component to support more efficiently the focal part of the reaching movement in weightless environment. Despite methodological differences with our study, Casellato et al. (2012) also reported whole-body forward displacement when performing unconstrained bimanual reaching (i.e., natural pace and uncontrolled accuracy). In line with their observations, our data may also support the existence of an “*oversimplification*” of postural control to perform a functional behavior when facing high spatiotemporal constraints of execution in microgravity. Moreover, unlike previous observations of Casellato et al. (2016) in long-term weightlessness, the postural strategy observed in the present study was not subjected to large inter-individual variability. Overall, individual trends for both focal and postural observations are clear and systematic (as illustrated in the Supplementary Figure 1). Remarkably, the stabilized motor features observed in microgravity in the present study appear close to those previously reported when participants performed the same task underwater with neutral buoyancy applied to body limbs (Macaluso et al., 2016). The following section discusses the behavioral analogies observed in both environments.

### Behavioral analogies between neutral buoyancy underwater and microgravity

As the present study is part of a scientific program studying human motor behavior in different force fields, the same participants were previously tested underwater in the same task for comparison purpose. Specifically, they were immersed in a prototypical submersible simulated space suit (AquaS environment; Macaluso et al., 2016), to apply neutral buoyancy at the level of body limbs. As in the present study conducted 2 years later, we did not find any significant evolution in the reported variables across the successive trials performed underwater. Rather, we observed immediate reorganizations at the earliest onset of exposure excluding the presence of adaptive processes during the experiment. As in MicroG, AquaS environment also implied initial exposure before data acquisition related to the installation of participants on the pointing structure. Thus, participants were submitted to global modifications of the force field applied to the whole-body before trial execution. This observation extends the hypothesis provided in section Prompt Reorganization of Motor Behavior in Microgravity. When participants accessed the new “unweighting” properties of a given environment before performing the first reaching movements, they could promptly reorganize their motor behavior. Most interestingly, the changes of focal and postural components of reaching movements in MicroG are close to those observed underwater in AquaS. Indeed, the increase of the relative arm deceleration duration and the decrease of trunk flexion when reaching far targets appear strikingly comparable (see the Supplementary Figure 2). In other words, the participants adopted analogous temporal structure of arm movements and almost similar postural strategy to perform whole-body reaching movements in these different environments. In so far as these two parameters are known to be gravity-dependent kinematic markers (see Introduction), and as AquaS and MicroG environments attempted to reproduce a weightless context, we hypothesize that these very close motor strategies would be mainly due to whole-body unweighting. It suggests that a fine control of neutral buoyancy underwater across the whole-body segments would tend to better simulate microgravity when considering the execution of sensorimotor tasks. Further studies are obviously required to challenge this hypothesis, especially to better investigate the effects of viscous force on motor control.

## Conclusion

The present study provides clear and original evidence that participants could successfully perform goal-directed whole-body reaching movements involving high spatiotemporal constraints in a novel environment, such as microgravity, by immediately reorganizing focal and postural control strategies compared to normogravity. Moreover, these substantial modifications occurred in motor planning at the very beginning of weightless exposure which strongly suggests that the effects of the absence of gravity were anticipated and integrated by CNS. Overall, our novel findings highlight that humans are able to maintain the performance of functional goal-directed whole-body actions in weightlessness in the standards of normogravity observations by successfully managing spatiotemporal constraints of execution in this unusual environment. Interestingly, our previous work reported similar kinematic features of whole-body reaching movements performed underwater when neutral buoyancy was rigorously applied at the level of each body limb (Macaluso et al., 2016). Therefore, this suggests that comparable initial state estimates and subsequent motor reorganizations could arise from unweighting the body at the level of body skin, muscles and joints, irrespective of the presence of gravity-related vestibular cues. Further experiments are of course mandatory to investigate this challenging hypothesis, which may be crucial for instance in astronauts training underwater, where gravitational field still acts at the level of the vestibular system.

## Ethics statement

This study was carried out in accordance with the recommendations of the ANSM (French National Agency for Biomedical Security) with written informed consent from all subjects. All subjects gave written informed consent in accordance with the Declaration of Helsinki. The protocol was approved by the CPP.

## Author contributions

TM designed and performed experiments, analyzed data and wrote the paper; CB wrote the paper; FB designed experiments and analyzed data; MM wrote the paper; PS designed and performed experiments, analyzed data; FS wrote the paper; JV wrote the paper; LB designed and performed experiments, wrote the paper.

### Conflict of interest statement

The authors declare that the research was conducted in the absence of any commercial or financial relationships that could be construed as a potential conflict of interest.
