# Supplementary material for: Sensorimotor Reorganizations of Arm Kinematics and Postural Strategy for Functional Whole-Body Reaching Movements in Microgravity
Source: Front Physiol. 2017 Oct 20;8:821. doi: 10.3389/fphys.2017.00821 (PMC5654841; doi:10.3389/fphys.2017.00821)
Supplement: Supplementary file 1 [file DataSheet1.pdf]

Supplementary Material

Sensorimotor reorganizations of arm kinematics and postural strategy for functional whole-body reaching movements in microgravity

T Macaluso, C Bourdin, F Buloup, M-L Mille, P Sainton, F Sarlegna, J-L Vercher, L Bringoux[*]

*** Correspondence:** Dr. Lionel Bringoux: lionel.bringoux@univ-amu.fr

1 Supplementary Figures

See Supplementary Figures above.

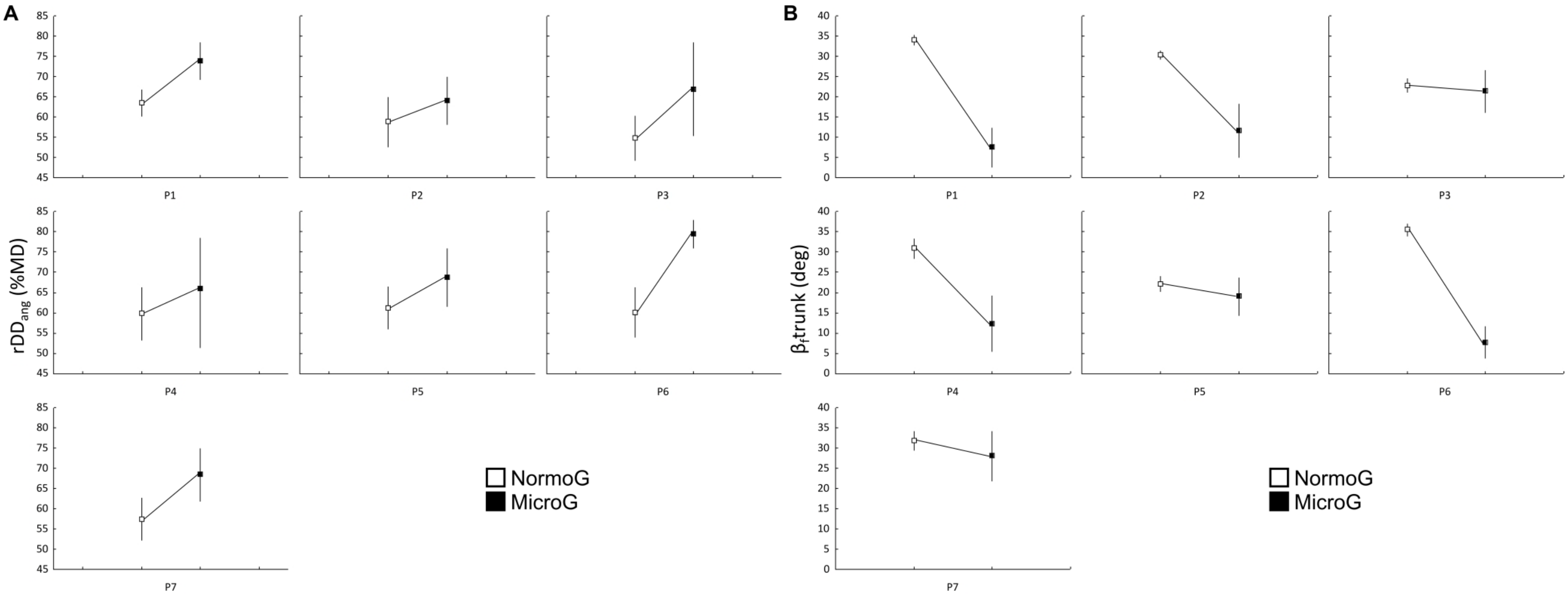

Supplementary Figure 1. (A) Mean relative angular deceleration duration (rDD_{ang}) and **(B)** mean final angular position of trunk relative to vertical ($\beta_f$trunk) for each participant as a function of Environment. Noticeably, the common changes in focal and postural organization relative to gravity environment for each participant illustrate the low between-subject variability in our observations.

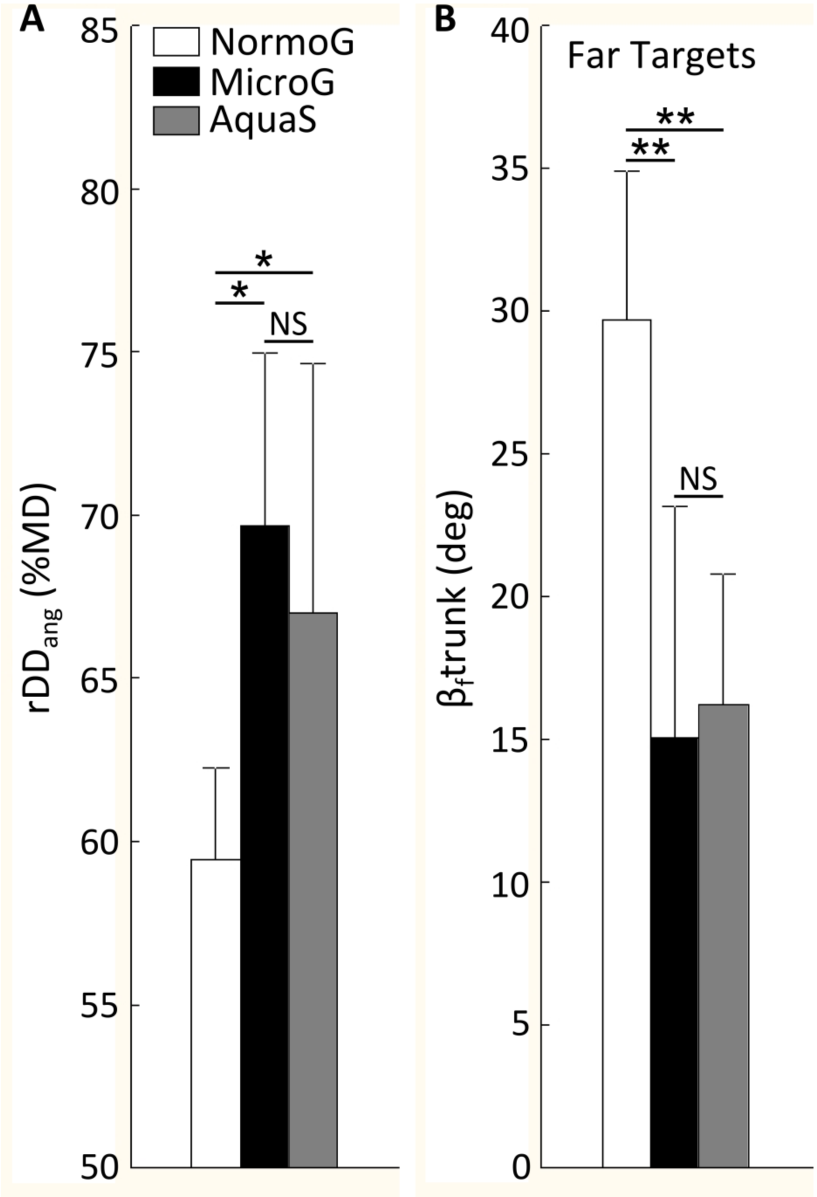

Supplementary Figure 2. (A) Mean relative angular deceleration duration (rDD_{ang}) and **(B)** mean final angular position of trunk relative to vertical ($\beta_f$trunk) as a function of Environment including AquaS data previously reported in our study underwater with neutral buoyancy applied to body limbs (Macaluso et al., 2016). Error bars represent standard deviation of the mean. $*p < .05$; $**p < .01$; NS: non-significant difference. This figure illustrates the main behavioral analogies between neutral buoyancy underwater (AquaS) and microgravity (MicroG) environments. In both studies, the participants adopted comparable temporal structure of arm movements and postural strategy to perform whole-body reaching movements in these different environments while distinguishing from normogravity observations.
